# Burden of self-reported acute gastrointestinal illness in China: a population-based survey

**DOI:** 10.1186/1471-2458-13-456

**Published:** 2013-05-08

**Authors:** Yan Chen, Wei-Xing Yan, Yi-Jing Zhou, Shi-Qi Zhen, Rong-Hua Zhang, Jiang Chen, Zhan-Hua Liu, Heng-Yi Cheng, Hong Liu, Sheng-Gang Duan, Zhen Lan, Ji-Chang Sun, Xing-Yong You, Jing-Guang Li, Yong-Ning Wu

**Affiliations:** 1 Key Laboratory of Food Safety Risk Assessment of Ministry of Health, China National Center for Food Safety Risk Assessment, 7 Panjiayuan Nanli, Chaoyang District, Beijing, 100021, China; 2 Jiangsu Provincial Center for Disease Control and Prevention, Nanjing, China; 3 Zhejiang Provincial Center for Disease Control and Prevention, Hangzhou, China; 4 Guangxi Regional Center for Disease Control and Prevention, Nanning, China; 5 Shanghai Municipal Center for Disease Control and Prevention, Shanghai, China; 6 Sichuan Provincial Center for Disease Control and Prevention, Chengdu, China; 7 Jiangxi Provincial Center for Disease Control and Prevention, Nanchang, China

## Abstract

**Background:**

Acute gastrointestinal illness (AGI) is an important public-health problem worldwide. Previous national studies of the incidence of AGI in China were performed decades ago, and detailed information was not available. This study therefore sought to determine the magnitude, distribution, and burden of self-reported AGI in China.

**Methods:**

Twelve-month, retrospective face-to-face surveys were conducted in 20 sentinel sites from six provinces between July 2010 and July 2011.

**Results:**

In total, 39686 interviews were completed. The overall adjusted monthly prevalence of AGI was 4.2% (95% confidence interval, 4.0–4.4), corresponding to 0.56 episodes of AGI per person-year. Rates of AGI were highest in children aged < 5 years. Healthcare was sought by 56.1% of those reporting illness. Of the cases who visited a doctor, 32.7% submitted a stool sample. The use of antibiotics was reported by 49.7% of the cases who sought medical care and 54.0% took antidiarrhoeals. In the multivariable model, gender, age, education, household type, residence, season, province and travel were significant risk factors of being a case of AGI.

**Conclusions:**

This first population-based study in China indicated that AGI represents a substantial burden of health. Further research into the specific pathogens is needed to better estimate the burden of AGI and foodborne disease in China.

## Background

Acute gastrointestinal illness (AGI) is an important cause of morbidity and mortality with global dimensions, affecting both developing and developed countries [1]. In developed countries foodborne diseases occur primarily as self-limiting AGI, characterized by diarrhoea or vomiting. Studies estimating the burden of AGI in a population provide the basis for assessing the burden of foodborne disease. Although AGI is very common in the community, not all cases present to the healthcare system, and not all cases that present are reported to national surveillance [2]. Because many episodes of AGI are not captured by routine data sources, the true burden of AGI was under-reported. To better determine the overall human health impact of foodborne diseases, cross-sectional surveys to quantify AGI have been conducted in a number of countries [3-5]. Knowledge of the magnitude of the disease burden is important for planning prevention strategies for gastroenteritis, especially foodborne diseases. The latest studies of the incidence of AGI in China were performed decades ago, and detailed information was not available [6]. China National Center for Food Safety Risk Assessment (previously known as the Food Safety Section of the National Institute for Nutrition and Food Safety, Chinese Center for Disease Control and Prevention) launched a national population-based study on the burden of AGI in 2010, and the data from a subsample of participants from Jiangsu province has recently been published [7]. The objectives of the present study were to estimate the magnitude, distribution, and burden of self-reported AGI in the Chinese population.

## Methods

### Study design and sample

**Figure 1:**
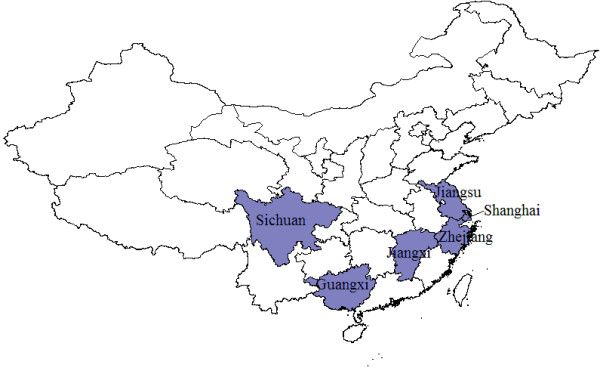
Map of the surveillance provinces.

Twelve-month, cross-sectional, face-to-face surveys were administered at 20 purposively-selected sentinel sites in six provinces in China between July 2010 and July 2011. These sentinel sites were geographically representative of China (e.g. urban/rural, south/north) (Figure 1). The sentinel sites were as follows: (1) Shanghai (Luwan and Qingpu district); (2) Jiangsu province (Xinqu district and Jiangyin county of Wuxi prefecture, Quanshan district of Xuzhou prefecture, Liyang county of Changzhou prefecture, Canglang district, Changshu county and Taicang county of Suzhou prefecture, Yizheng county of Yangzhou prefecture, and Hailing district of Taizhou prefecture); (3) Zhejiang province (Xihu district and Chunan county of Hangzhou prefecture, Tongxiang county of Jiaxing prefecture, and Deqing county of Huzhou prefecture); (4) Jiangxi province (Donghu district of Nanchang prefecture); (5) Guangxi province (Quanzhou county of Guilin prefecture, Lingshan county of Qinzhou prefecture, and Pinguo county of Baise prefecture); and (6) Sichuan province (Panzhihua prefecture). The sentinel sites were selected based on their suitability, willingness of local authorities and feasibility of completing the studies. The sentinel sites represent approximately 1.0% of the total permanent resident population (1332810869) in China in 2010. Each sentinel site was divided into several blocks in terms of administrative division and population distribution. In total, we generated 2806 blocks from the 20 sentinel sites; from each block, city block in urban areas or village in rural areas was randomly selected. Households were randomly selected from each city block or village using the lottery method and the number of households surveyed was proportional to population size. In each household, the person who was next to celebrate a birthday was selected to participate in the survey. Up to three attempts were made to contact the selected individual at different times of the day to complete the survey. If the selected individual declined or no one lived in the house, the neighbouring house was selected as replacement. Proxy respondents were used for all children < 12 years, and for individuals aged 12–18 years, at the discretion of the parent or guardian. Written and informed consent was obtained from all respondents before the interview. All interviews were conducted in Chinese. A target sample size of 2850 interviews per month in the surveillance areas was calculated to detect a prevalence of 5%, with a 5% level of precision and a 0.8% allowable error (Epi Info v. 3.0.2, CDC, USA). For each sentinel site, 100 to 300 interviews were completed each month, and the target number of completed interviews was homogeneously distributed throughout the duration of the study period, in 12 monthly waves. This study received ethics approval from the Committee on Human Experimentation of National Institute for Nutrition and Food Safety, Chinese Center for Disease Control and Prevention.

### Data collection

Household interviews were conducted by trained health workers using a standard questionnaire. The questionnaire was validated and developed specifically for the purposes of this study. The respondents were asked whether they had experienced diarrhoea or vomiting in the 4 weeks before the interview. Additional questions were asked about demographic characteristics. If the respondent reported diarrhoea or vomiting in the relevant timeframe, he or she was asked for more details regarding symptoms and timing, perceived causes of their illness, travel history (if they traveled to other city/province), the use of medical consultations and treatment, admission to hospital and whether a stool sample was sent for diagnostic purposes, the social and economic impact of illness, and whether other members in the household had experienced diarrhoea or vomiting concurrently. Duration was collected for diarrhoea but not for AGI or vomiting. If AGI was defined by presence of diarrhoea (rather than vomiting), then it would seem that for AGI based on diarrhoea, the duration would be equivalent [8,9]. The definition of urban and rural populations were based on the Regulation on Statistical Classification of Urban and Rural Areas (China) issued by the National Bureau of Statistics in 2008 [10]. Additional file 1 is the translated questionnaire used in the survey.

### Case definition

AGI was defined as diarrhoea of ≥ 3 loose stools in a 24-hour period or significant vomiting with at least one other symptom (abdominal pain/cramps, fever), but excluding those (a) with Crohn’s disease, irritable bowel syndrome, colitis, diverticulitis of large intestine, or another chronic illness with symptoms of diarrhoea or vomiting, or (b) who report their symptoms were due to pregnancy, excess alcohol, chemotherapy/radiotherapy, drugs, or food allergy. A 7-day symptom-free interval was defined to distinguish multiple episodes. In case of multiple episodes, only the most recent episode of AGI was described.

### Statistical analysis

All questionnaires were reviewed for legibility and completeness. EpiData version 3.1 (EpiData Association, Odense M, Denmark) was used for data entry in each province. All the data analyses were performed using the SPSS version 16.0 (SPSS Inc., Chicago, IL, USA). The response rate was calculated as the number of completed surveys divided by the number of households visited. The monthly prevalence of AGI was calculated by dividing the number of respondents reporting AGI in the 4 weeks prior to the interview by the total number of respondents. The point prevalence of AGI was obtained as the proportion of cases with symptoms on the day of the interview. The prevalence of seeking medical care for AGI was calculated as the number of respondents who saw a doctor for his/her disease divided by the number of AGI cases. The proportion of stool sample submission was the proportion doctors taking stool sample for laboratory testing from patients with diarrhoeal symptoms. For incidence rate calculations, respondents reporting multiple episodes were counted as a single episode. The incidence rate of AGI per person-year was calculated using the terminology and formulae outlined by Rothman and Greenland [11,12].

These estimates were adjusted for known differences between the survey sample and the target population by weighting for age, sex, residence and provinces using the sixth national population census as the reference population [13]. Because of the weighting, it is not possible to present the actual numbers on which percentage estimates are based. The association between demographic factors and the occurrence of AGI was tested using the *x*^2^ test. When calculating the mean duration of diarrhoea, persons with ongoing AGI symptoms were not excluded. Mean duration of diarrhoea in different age groups was compared by analysis of variance (ANOVA). P-values less than 0.05 were considered statistically significant. Multivariable logistic regression was used to estimate odds ratios (ORs) for each of the geographic and demographic variables. Weighting was not included in the logistic regression since the purpose of the analysis was to identify relative ORs among risk factors. In multivariable analysis, to determine the variables in the model, we used the forward elimination method. Explanatory variables tested were gender, age, education, occupation, household size, household type, residence, season, province and travel history. Variables with a p-value less than 0.05 were included in the final model.

To facilitate international comparisons, Majowicz et al. proposed a minimum set of reported results and a standard symptom-based case definition for AGI of ≥ 3 loose stools or any vomiting in a 24-hour period, excluding those (a) with cancer of the bowel, irritable bowel syndrome, Crohn's disease, ulcerative colitis, cystic fibrosis, coeliac disease, or another chronic illness with symptoms of diarrhoea or vomiting, or (b) who report that their symptoms were due to drugs, alcohol, or pregnancy [9]. We reported the minimum set of results, using the study definition to allow future international comparisons.

## Results

### Response rate and representativeness of the sample

The Jiangsu, Zhejiang, Shanghai and Guangxi studies were conducted from July 2010 to June 2011 and surveyed 10959, 9548, 7176 and 7138 residents, respectively. The Sichuan and Jiangxi studies were conducted from August 2010 to July 2011 and surveyed 3078 and 1787 residents, respectively. Complete interviews were conducted with 39686 of the 42470 individuals contacted to participate in this study, yielding an overall response rate of 93.4% (range 84.1% to 99.4%). In general, survey respondents were older, more educated and more likely to be male than residents (Table 1).

**Table 1:** Characteristics of respondents and weighted monthly prevalence of reporting acute gastrointestinal illness in the 4 weeks prior to interview in China, 2010–2011

**Variable**	**Chinese general population (%)**	**Survey Respondents (%)**	**Monthly Prevalence of AGI**		**P-value**
			**%**	**(95% CI)**	
Gender (n = 39681)					< 0.01
Male	51.2	60.3	3.9	(3.6–4.2)	
Female	48.8	39.7	4.6	(4.2–4.9)	
Age (years) (n = 39678)					< 0.01
0–4	5.7	2.6	12.6	(11.1–14.1)	
5–14	10.9	2.6	2.9	(2.3–3.4)	
15–24	17.1	4.4	4.0	(3.5–4.5)	
25–44	33.1	35.0	3.3	(3.0–3.7)	
45–64	24.3	38.9	3.9	(3.5–4.3)	
≥ 65	8.9	16.4	5.2	(4.5–6.0)	
Ethnic group (n = 39650)					0.025
Han	91.6	92.6	4.3	(4.1–4.5)	
Minority	8.4	7.4	3.4	(2.7–4.1)	
Education (n = 39664)					< 0.01
Preschool children	6.8	3.2	10.5	(9.3–11.6)	
Illiterate	4.7	6.9	6.2	(5.1–7.3)	
Primary school	26.8	24.4	4.1	(3.7–4.5)	
Secondary school	38.9	34.4	4.0	(3.6– 4.4)	
High school and above	22.9	31.1	2.9	(2.7–3.2)	
Occupation (n = 39673)					< 0.01
Labourer	NA	13.4	3.1	(2.6–3.6)	
Services	NA	3.3	2.5	(1.8–3.3)	
Administrator/Director	NA	2.4	3.4	(2.3–4.5)	
Office staff	NA	4.8	2.6	(1.9–3.2)	
Professional	NA	4.4	3.5	(2.7–4.3)	
Farmer	NA	39.4	4.8	(4.3–5.2)	
Self-employed	NA	4.8	4.3	(3.4–5.2)	
Retired	NA	15.1	2.8	(2.2–3.4)	
Unemployed	NA	3.3	4.5	(3.5–5.5)	
Too young to work (including students)	NA	6.5	6.1	(5.6–6.7)	
Others	NA	2.6	2.8	(1.8–3.9)	
Total family income per year (n = 39546)^a^					< 0.01
0–29999 yuan	NA	34.1	5.2	(4.8–5.6)	
30000–79999 yuan	NA	49.2	3.6	(3.3–3.8)	
≥ 80000 yuan	NA	16.7	4.0	(3.5–4.5)	
Household size (number of person) (n = 39677)					0.041
1–2	38.9	21.5	3.7	(3.2–4.2)	
≥ 3	61.1	78.5	4.3	(4.1–4.5)	
Household type (n = 39666)					< 0.01
No residents < 18 years	NA	49.8	3.5	(3.2–3.7)	
At least one resident < 18 years	NA	50.2	4.7	(4.4–5.0)	
Residence (n = 39686)					< 0.01
Urban	50.3	37.5	3.5	(3.3–3.8)	
Rural	49.7	62.5	5.1	(4.8–5.4)	
Province (n = 39686)					< 0.01
Shanghai	1.7	18.1	1.2	(0.8–1.6)	
Jiangsu	5.9	27.6	4.7	(4.3–5.2)	
Zhejiang	4.1	24.1	3.1	(2.7–3.5)	
Jiangxi	3.3	4.5	5.7	(4.7–6.6)	
Guangxi	3.5	18.0	3.6	(3.1–4.1)	
Sichuan	6.0	7.8	5.3	(4.9–5.8)	
Travel (n = 39675)					< 0.01
Yes	NA	4.0	8.6	(7.1–10.0)	
No	NA	96.0	4.0	(3.8–4.2)	

CI, Confidence interval; NA, Not available.

^a^ The average yearly income in cities and towns was 36539 yuan in 2010, while the average yearly income in the private sector was 20759 yuan [14].

### Frequency and distribution of AGI

Of the 39686 individuals included in the study, 1376 (3.5%; 95% CI 3.3–3.7) reported having experienced symptoms of gastroenteritis in the previous 4 weeks. Of these respondents, 109 were identified as non-infectious cases and included in the non-case category, leaving 1267 respondents to be identified as cases. After excluding these respondents, an overall prevalence of self-reported AGI in the 28 days prior to the interview, adjusted for age, sex, residence and province was 4.2% (95% CI 4.0–4.4). This represents an average of 0.56 (95% CI 0.56–0.57) episodes of AGI per person-year. A total of 118 cases had symptoms of AGI on the date of the interview, yielding an unadjusted point prevalence of 0.3% (95% CI 0.2–0.4) and age, sex, residence and province adjusted prevalence of 0.4% (95% CI 0.4–0.5).

**Figure 2:**
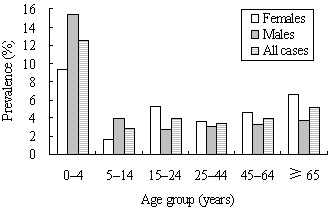
Monthly prevalence of acute gastrointestinal illness, by age and gender, in the 4 weeks prior to interview in China, 2010 to 2011 (n = 39686).

**Figure 3:**
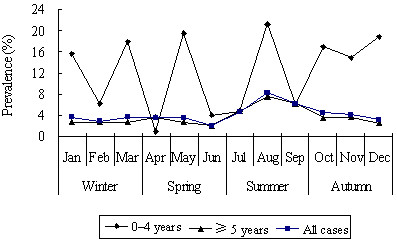
Monthly prevalence of acute gastrointestinal illness, by study month, in the 4 weeks prior to interview in China, 2010 to 2011 (n = 39686).

Estimates of the monthly prevalence of AGI by demographic characteristics are reported in Table 1. The monthly prevalence was significantly higher in females than in males (4.6% vs. 3.9%, p < 0.01). Children aged 0–4 years had the highest prevalence of AGI. This peak was observed in both males and females, but the increase was greater among males than females (Figure 2). The prevalence of AGI was highest in preschool children and declined with the increase of educational levels. Compared with the other income groups, the prevalence of AGI was significantly higher in households with income lower than 30000 yuan (p < 0.01). Of the respondents, 78.5% households had ≥ 3 household members, and the prevalence of AGI was significantly higher in these larger households (4.3%). Respondents living in a household with at least one person aged < 18 years were more likely to have experienced AGI (p < 0.01). There was a difference in prevalence of AGI between the urban (3.5%) and rural areas (5.1%) (p < 0.01). There was a seasonal difference of the prevalence of AGI: summer had the highest prevalence of AGI, followed by autumn (p < 0.01) (Figure 3). The prevalence of AGI varied by provinces, with the highest in Jiangxi (5.7%), and the lowest in Shanghai (1.2%) (p < 0.01). The percentage of respondents reporting AGI was higher in those who had traveled (8.6%) than those who had not (4.0%) (p < 0.01).

### Multivariable analysis

In multivariable regression analysis, gender, age, education, household type, residence, season, province and travel were more likely (p < 0.05) associated with the occurrence of AGI (Table 2). The odds ratio of AGI was 2.5 times higher in summer than in winter. History of travelling to other city/province was positively associated with AGI.

**Table 2:** Final multivariable model of risk factors associated with acute gastrointestinal illness in the 4 weeks prior to interview in China, 2010–2011

**Variable**	**OR**	**95% CI**	**P-value**
Gender			
Male	Ref.	Ref.	Ref.
Female	1.19	1.06–1.34	< 0.01
Age (years)			0.02
0–4	2.82	1.31–6.05	0.01
5–14	1.12	0.77–1.64	0.55
15–24	1.20	0.91–1.58	0.20
25–44	0.90	0.77–1.05	0.18
45–64	Ref.	Ref.	Ref.
≥ 65	0.90	0.74–1.08	0.26
Education			< 0.01
Preschool children	1.10	0.53–2.29	0.80
Illiterate	1.54	1.21–1.95	< 0.01
Primary school	1.04	0.89–1.23	0.62
Secondary school	Ref.	Ref.	Ref.
High school and above	0.78	0.66–0.91	< 0.01
Household type			
No residents < 18 years	0.86	0.75–0.97	0.02
At least one resident < 18 years	Ref.	Ref.	Ref.
Residence			
Urban	0.86	0.74–0.99	0.04
Rural	Ref.	Ref.	Ref.
Season			< 0.01
Winter	Ref.	Ref.	Ref.
Spring	0.92	0.76–1.12	0.40
Summer	2.51	2.14–2.95	< 0.01
Autumn	1.34	1.12–1.60	< 0.01
Province			< 0.01
Shanghai	0.34	0.27–0.43	< 0.01
Jiangsu	Ref.	Ref.	Ref.
Zhejiang	0.65	0.55–0.76	< 0.01
Jiangxi	1.00	0.75–1.33	0.99
Guangxi	0.62	0.52–0.74	< 0.01
Sichuan	1.21	0.99–1.47	0.06
Travel			
Yes	2.44	1.96–3.03	< 0.01
No	Ref.	Ref.	Ref.

OR, Odds ratio; CI, Confidence interval.

### Symptoms and severity

Among the 1267 cases, 1260 (99.4%) reported suffering from diarrhoea, 251 (19.8%) reported suffering from vomiting and 244 (19.3%) cases experienced both symptoms. Of the 1260 cases with diarrhoea, 34 (2.7%) had bloody diarrhoea. Among the 1109 cases who reported diarrhoea within the 4-week period and who did not report being ill at the time of interview, the average duration of illness was 1.8 days (range 1 –20). Children in the 0–4 year group had a significantly longer duration of diarrhoea (3.0 days) than other age groups (p < 0.01). On the worst day of symptoms, cases reported diarrhoea an average of 4 times (range 3–15 times) and vomiting an average of 3 times (range 1–10 times). Of the 1267 cases with AGI, 51 (4.0%) reported more than one episode in the previous 28 days.

### Suspected cause of illness

Cases were asked to report what they believed was the cause of their illness. The suspected causes were: consuming contaminated food (36.5%) or water (4.0%), and contact with an infected person (0.6%). Of those who suspected that their illness were due to eating contaminated food, 57.7% (267/463) thought it was food consumed at home.

### Healthcare and impact

Healthcare-seeking behaviour, medication use and the source of the medication are reported in Table 3. In total, 48.8% of the cases visited a doctor. After adjusting for age, sex, residence and province the prevalence of seeing a doctor was 56.1% (95% CI 53.6–58.6). Healthcare-seeking behaviour varied by age; the adjusted prevalence of seeking health care for AGI was the highest in children < 5 years of age (73.6%). Among those who saw a doctor, 33.6% reported having provided a stool sample for laboratory testing. After adjusting for age, sex, residence and province the proportion of stool sample submission among those who saw a doctor was 32.7% (95% CI 29.6–35.8).

**Table 3:** Frequency of hospital consultation and medicine use by cases of acute gastrointestinal illness (n = 1267) in the 4 weeks prior to interview in China, 2010–2011

**Variable**	**No. of case (%)**
Sought medical care (n = 1263)	
Yes	616 (48.8)
No	647 (51.2)
Submit a stool sample (n = 614)	
Yes	206 (33.6)
No	408 (66.4)
Take medicine (n = 1267)	
Yes	1070 (84.5)
No	197 (15.5)
Type of medicine (n = 1069)^a^	
Antibiotics	630 (58.9)
Antidiarrhoeals	684 (64.0)
Analgesics	49 (4.6)
Antipyretics	22 (2.1)
Antacids	16 (1.5)
Other	41 (3.8)
Unknown	49 (4.6)
Location of medicine purchase (n = 1069)^a^	
Family medicine chest	298 (27.9)
Hospitals with prescription	607 (56.8)
Pharmacy	234 (21.9)
Other	21 (2.0)

^a^ Because some people took more than one type of medication and some cases visited more than one location, so the total percentage may exceeds 100%.

In total, 84.5% cases took medications to treat or relieve symptoms for their illness. Half of those with AGI were estimated to have taken antibiotics, while 54.0% reported taking antidiarrhoeals. Of those who took medicine, only 56.8% reported that the medication was prescribed by a doctor, 27.9% obtained medicine from the family medicine chest, 21.9% bought medicine from a pharmacy without prescription. However, 27.6% cases reported taking more than one type of medication to treat or relieve symptoms.

Overall, 9.3% of those with AGI reported taking time off work. The mean number of days taken off work was 2.4 days (range 1–12 days). Of those with AGI, 1.4% reported not being able to go to school. The mean number of days of school absenteeism were 1.8 (range 1–4 days). Of persons with AGI, 3.9% (49/1263) reported that at least one other person living in the same household had suffered from diarrhoea or vomiting during the 28 days prior to the interview.

### Standard case definition comparison

To facilitate international comparison, the results of the study were summarized in Table 4, using the standard symptom-based case definition for AGI proposed by Majowicz et al. [9].

**Table 4:** **Descriptive statistics of acute gastrointestinal illness following the proposed standard case definition of acute gastrointestinal illness**^**a **^**in China, 2010–2011**

	
Incidence per person-year (95% CI)	0.57 (0.56–0.57)
Incidence per person-year in males	0.53
Incidence per person-year in females	0.61
Mean age of cases (years)	44
Mean duration of illness (days)^b^	2.1
Cases with bloody diarrhoea (%)	2.66
Cases who sought medical care (%)	55.94
Cases submitting a stool sample for testing (%)	18.14
Cases with respiratory symptoms (%)^c^	–
Cases with symptoms still ongoing at time of interview (%)	9.3

^**a**^ Acute gastrointestinal illness was defined as diarrhoea of ≥ 3 loose stools or any vomiting in a 24-hour period.

^b^ Information on the duration of vomiting was not collected in the survey. Mean duration calculated by averaging the duration of illness among those had diarrhoea, regardless of whether they were still ongoing at the time of data collection.

^c^ Data not collected. Survey respondents were not asked about respiratory symptoms.

## Discussion

This is the first nationwide survey on AGI in the general population in China. Our results show that AGI represent a substantial burden in China. Based on the estimates of 0.56 AGI episodes per person-year and 56.1% of cases seeking medical care for AGI, 748 million cases of AGI and 420 million medical consultations occur each year throughout the country. The mean number of days taken off work was 2.4 days. If extrapolated to the population, this equates to about 167 million working days lost. Based on an incidence of 0.56 AGI episodes per person-year, by extrapolating the average of the proportion of AGI that is foodborne published from the USA (25%), England and Wales (26%) and Australia (32%) [15-18], it is estimated that there are 209 million cases of foodborne disease in China in one year. As the actual fraction of AGI due to foodborne disease is unknown, the extrapolation is merely to bring in context from other studies.

It is difficult to compare AGI rates between studies due to the use of different case definitions and study designs. Using the standard symptom-based case definition for AGI proposed by Majowicz et al. [9], we obtained similar results as compared with those using the chosen definition in our primary analysis. We thus reported the suggested minimum set of results in this article, in order to facilitate international comparisons. Overall the estimated rate of AGI in China falls within the range of incidence reported in similar retrospective studies performed in other countries [4,5,19-23]. The AGI incidence estimated in the Chinese population was lower when compared with Australia (0.8), Poland (0.9), Italy, USA and Canada (1.0), New Zealand (1.1), Denmark (1.4), and higher compared with France (0.3), Ireland and Japan (0.4). The observed differences between countries could be related to different exposures based on lifestyle, such as food consumption habits. The estimates of disease burden in the community differ substantially between retrospective and prospective study designs even when using identical case definitions. Prospective cohort studies tend to give lower rates with a rate of 0.28 episodes per person-year in The Netherlands [24] and a rate of 0.27 in the United Kingdom [25]. This discrepancy was once attributed to telescoping, with respondents remembering disease episodes as having occurred more recently than they actually did. However in other studies [26-28], it is reported that the shorter 7-day recall period yields significantly greater annual estimates compared to the longer 30-day recall period, which is contrary to telescoping resulting in the true burden of disease being underestimated. The number of episodes per year based on the point prevalence estimate (0.4%) in the present study was 0.81, much higher than the estimate (0.56) based on the monthly prevalence. These differences also highlight the need for further standardization of the survey methods used.

This study found a higher rate of AGI in females and in children < 5 years, which has also been the case in other studies [12,29]. Handling food and caring for children may underlie this pattern, bringing females more frequently in contact with enteric pathogens than males. It is likely that the behaviour of young children may increase their exposure to pathogens via person-to-person and environmental transmission. Identification of groups of people vulnerable to gastroenteritis is useful to guide resource allocation. Seasonality of AGI was observed in this study, with a peak of incidence during the summer months as in Australia [30]. These months are traditionally associated with increased rates of laboratory proved *Salmonella* and *Vibrio* gastroenteritis [31,32]. It should be noted that although Australia has a summertime seasonality, because it is in the southern hemisphere, this is really a wintertime seasonality typical of norovirus transmission. Further research is needed to understand why this may be different in China.

In our study, people who were less wealthy and who had a lower level of education were more likely to report AGI, a finding different from the United States and Australia, but similar to Malaysia and Malta [30,33-35]. This may be due to the different lifestyle behaviours in this group such as health habits, eating-out patterns and awareness of hygienic conditions. However, the multivariate analysis showed that the variation across income groups was not significant.

The proportion of cases reporting that consuming contaminated food (36.5%) was a cause of their illness was lower than those reported in Hong Kong (45.0%) but much higher than the proportion in Ireland (18.5%) [29,36]: an indication of some uncertainty. In practice, people seldom actually know the cause of their AGI, although they often attribute it to food. However, if there is no better way to get the foodborne proportion of AGI, this subjective proportion may be used to estimate the incidence of foodborne AGI, similar to Evans et al. [37].

The proportion of cases who visited a doctor (56.1%) and the level of medicine use (89.2%) found in this study were higher than the proportions observed in other countries cited above. These differences may reflect important differences in healthcare systems in the studied countries. However, the fact that a relatively high proportion of cases visited a doctor may also suggest that this study is primarily capturing the severe cases. Antibiotics are very rarely for the treatment of AGI. However, their use was reported by 49.8% of those with AGI, much higher when compared with other countries [4,5,19,22,28,38]. Inappropriate usage of antimicrobial agents to treat humans is a major problem in China, as many Chinese receive antibiotics before they are assessed by a doctor or before diagnostic tests are performed [32,39,40]. If there are 748 million episodes of AGI each year in China, the number of AGI cases who are treated with antibiotics may be 372 million. This is of major concern, particularly in view of the increase in antibiotic resistant pathogens and the potential complications arising from taking antibiotics. It is important to understand the factors influencing the prescription and use of antibiotics, and to promote appropriate usage of antibiotics by people in China.

Of the respondents with AGI who visited a doctor, about one in third provided a stool sample, which was higher than in other countries [4,5,20,22,23]. If there are 420 million medical consultations each year in China, the number with stool exam is 137 million. This provides a rich opportunity to document pathogen-specific illness rates. However, in China, if a stool specimen is received, it is routinely tested for white blood cells but not cultured for pathogen [41]. Although most of the stool exams are for white blood cells rather than culture, the fact that the practice of seeking health care and submitting a stool sample is important. As cheap and fast non-culture diagnostic tests are disseminated, their use in China could revolutionize our approach to foodborne disease surveillance. The potential for this should be further studied, particularly since there is a summertime (bacterial pathogen) seasonality.

The main limitation in this study was recall bias, which may arise because individuals with a particular condition are likely to remember their experiences differently from those who are not similarly affected. Attempts were made to reduce this bias by asking the actual date of onset, which gives more accurate results. Another limitation was caused by the potential co-existence of AGI symptoms with respiratory symptoms, both of which can be caused by either enteric disease or respiratory infection. In the present study, respondents were not asked about respiratory symptoms or illnesses; hence, AGI associated with respiratory illness was not excluded from our analysis, which means that the study may have in part also measured symptoms caused by non-intestinal infections [42]. When estimating the burden of foodborne disease based on data from population studies, it is necessary to consider this issue. The advantage of this study is that we used a face-to-face interview, which may reach the potential participants who do not have access to a telephone; also the respondents were selected by the "next birthday" method, which was easy to operate and successfully in randomly selecting respondents. The response rate was quite high in this study compared to surveys from other countries cited above.

Estimating the frequency of AGI cases in a population is an important issue in evaluating the burden of foodborne disease. Although different sources of limitation could have restricted the efficacy of our study in providing the burden of AGI in the Chinese population, our study provides a contribution to a comprehensive estimate of the global burden of foodborne disease. The data regarding healthcare-seeking behaviour and the proportion of patients who have a stool sample submitted for analysis in particular, will help fill a gap in the efforts to estimate the size of each layer in the surveillance pyramid for AGI in China. Although the data obtained are not pathogen specific, the dataset can assist in the efforts to calculate the cost and disease burden of AGI in China. Due to the methodological challenges inherent in the cross-sectional study design, the results are not conclusive; nevertheless, they suggest that a substantial amount of AGI occurs which leaves room for preventive measures, e.g. in terms of efforts to prevent foodborne disease outbreaks, improve food safety and improve hygiene.

## Conclusions

We conclude that acute gastrointestinal illness (AGI) represents a substantial burden on health in China. Further research into the pathogen-specific burden of AGI is necessary to better estimate the burden of AGI and foodborne disease in China, and more efforts will focus on the respiratory syndrome and the recall period.

## Abbreviations

AGI: Acute gastrointestinal illness; ANOVA: Analysis of variance; CI: Confidence interval; NA: Not available; OR: Odds ratio

## Competing interests

The authors declare that they have no conflict of interest.

## Authors’ contributions

YC performed design, analysis and interpretation of data, drafting the first version of the manuscript. WXY and YNW were both involved in the coordination of the project, and contributed to drafting subsequent versions of the manuscript. YJZ, SQZ, RHZ, JC, ZHL, HYC, HL, SGD, ZL, JCS, XYY assisted in the design and coordination of the project. JGL contributed to drafting subsequent versions of the manuscript. All authors have read and approved the final manuscript.

## Pre-publication history

The pre-publication history for this paper can be accessed here:

http://www.biomedcentral.com/1471-2458/13/456/prepub

## Supplementary Material

Additional file 1 Study questionnaire. Click here for file
