# Supplementary material for: Burden of self-reported acute gastrointestinal illness in China: a population-based survey
Source: BMC Public Health. 2013 May 8;13:456. doi: 10.1186/1471-2458-13-456 (PMC3655923; doi:10.1186/1471-2458-13-456)
Supplement: Additional file 1 — Study questionnaire. [file 1471-2458-13-456-S1.doc]

**Study Questionnaire**

Date of interview Years/Months/Days

**Section one: Basic information**

1. Sentinel site code

2. Person code

3. If the questionnaire being answered by the selected respondent him or herself?

[1] Yes [2] No

4. How many people are usually live in this household?

4a. How many are <18 years of age?

5. Total family income per year yuan

6. Home address

Province Prefecture District/County

Township/Street Village/Community

7. Residence [1] Urban [2] Rural

8. Telephone

9. What is your (your child’s) name?

10. Gender [1] Male [2] Female

11. Age years

12. Ethnic group [1] Han [2] Manchu [3] Mongol [4] Hui [5] Tibetan [6] Miao

[7] Zhuang [8] Uyghur [9] Other

13. Education

[1] Preschool children [2] Illiterate [3] Primary school [4] Secondary school

[5] High school [6] Technical secondary school and junior college

[7] University [8] Postgraduate

14. Occupation

[1] Teacher [2] Worker [3] Migrant labourer [4] Peasant [5] Herdsman

[6] Fisherman [7] Housekeeper [8] Employed persons in catering services

[9] Business service personnel [10] Administrator/Director [11] Office staff

[12] Research specialist staff [13] medical personnel [14] Self-employed

[15] Retired [16] Unemployed [17] Too young to work (including students)

[18] Other

15. During the past two weeks did you (your child) travel outside of the district/county where your household resides? [1] Yes [2] No

**Section two: Symptoms**

Acute gastrointestinal illness was defined as diarrhoea of ≥ 3 loose stools in a 24-hour period or significant vomiting with at least one other symptom (abdominal pain/cramps, fever), but excluding those (a) with Crohn’s disease, irritable bowel syndrome, colitis, diverticulitis of large intestine, or another chronic illness with symptoms of diarrhoea or vomiting, or (b) who report their symptoms were due to pregnancy, excess alcohol, chemotherapy/radiotherapy, drugs, or food allergy.

16. During the past 4 weeks, have you (your child) suffered from diarrhoea?

[1] Yes [2] No

16a. If yes, how many times per day?

17. Have you (your child) suffered from bloody diarrhoea? [1]Yes [2] No

17a. If yes, how much blood was there in your (your child’s) stool?

[1] Just a little blood on the toilet paper

[2] Some blood mixed with the stool

[3] So much blood that the stool was almost entirely blood

18. Have you (your child) suffered from vomiting? [1] Yes [2] No

18a. If yes, how many times per day?

If you have vomiting, did you (your child) also experience any of the following symptoms?

18b. Diarrhoea [1] Yes [2] No

18c. Abdominal pain/cramps [1] Yes [2] No

18d. Fever [1] Yes [2] No

19. Are you (your child) still suffering from any of the above symptoms?

[1] Yes [2] No

20. How long did the diarrhoea last? Days Hours

21. Cause of the illness

21a. What do you think caused your (your child’s) symptoms?

[1] Food poisoning [2] Person-to-person [3] Contaminated water

[4] Animal contact [5] Other [6] Unknown

21b. Other, please specify

21c. Do you think that your (your child’s) symptoms are caused by the following condition, such as Crohn’s disease, irritable bowel syndrome, colitis, 0000000000000000000000000000000000000000000000000000000000000000000000000000000000000000000000000000000000000000000000000 9]以下情况 00000000000000000000000000000000000000000000000000000000000000000000000000000000000000000000000000000000000000000000000diverticulitis of large intestine, pregnancy, excess alcohol, chemotherapy/radiotherapy, medication, food allergy? [1] Yes [2] No

22. A 7-day symptom-free interval was defined to distinguish multiple episodes. Judged by the interviewer, how many episodes of acute gastrointestinal illness did the respondent have during the past 4 weeks?

**Section three: Suspected food**

23. If food poisoning, which food you think was most suspected to cause your (your child’s) symptoms?

23a. Type of food

[1] Meat and meat products [2] Milk and dairy products

[3] Eggs and egg products [4] Fishery products [5] Cereals and cereal products

[6] Beans and bean products [7] Vegetable Oil [8] Fruits and vegetables

[9] Other [10] Unknown

24. If food poisoning, where do you think you (your child) got the food that caused your (your child’s) symptoms?

[1] Own home [2] Private house (excluding own home) [3] Hotel/Restaurant

[4] Fast food service [5] Food supermarket [6] Street vender [7] Takeaway

[8] School cafeteria [9] Company cafeteria [10] Food service on construction sites

[11] Other [12] Unknown

24a. Other, please specify

**Section four: Medical treatment**

25. As a result of this illness for how many times did you (your child) visit a doctor?

(enter ‘0’ if none)

26. If visited a doctor, did you visit a doctor because you wanted diagnosis and treatment or required certificate for work? [1] Yes [2] No

26a. Wanted diagnosis and treatment [1] Yes [2] No

26b. Required certificate for work [1] Yes [2] No

27. As a result of this illness for how many days were you (your child) hospitalized?

(enter ‘0’ if none)

28. Were you (your child) asked to submit a stool sample? [1] Yes [2] No

28a. The result of the stool sample (enter the etiology being identified by the laboratory, if not sure, enter ‘unknown’)

29. Did you (your child) take any medications for this illness? [1] Yes [2] No

29a. Pharmacy [1] Yes [2] No

29b. Hospitals with prescription [1] Yes [2] No

29c. Family medicine chest [1] Yes [2] No

29d. Other, please specify

30. How many days were medications taken for?

31. Name of the medication(s) (enter ‘unknown’ if not sure)

Type of medicine

31a. Antibiotics [1] Yes [2] No

31b. Antidiarrhoeals [1] Yes [2] No

31c. Analgesics [1] Yes [2] No

31d. Antipyretics [1] Yes [2] No

31e. Antacids [1] Yes [2] No

31f. Other [1] Yes [2] No

31g. Unknown [1] Yes [2] No

**Section five: Social and economic impact of illness**

32. Did this illness require you (your child) to miss work or school/college?

[1] Yes [2] No

32a. Days missed from work (enter ‘0’ if none)

32b. Days missed from school/college (enter ‘0’ if none)

33. Did anyone else in your household have similar symptoms? If yes, how many?

(enter ‘0’ if none)

Interviewer Assessor
